# An unusual origin of the double left testicular artery in a male cadaver: a case report

**DOI:** 10.1186/1752-1947-6-267

**Published:** 2012-08-31

**Authors:** Branislav Filipovic, Lazar Stijak, Branka Filipovic

**Affiliations:** 1 Institute of Anatomy “Niko Miljanic”, University of Belgrade, Faculty of Medicine, 4/2 Dr Subotica, 11000, Belgrade, Serbia; 2 Department of Gastroenterohepatology, Clinical and Hospital Center “Bezanijska Kosa”, Autoput s/n, 11080, Belgrade, Serbia

## Abstract

**Introduction:**

Variations in the number and course of the testicular arteries, often coexisting with variations of the other branches arising from the abdominal aorta, are still reported to be of interest to urology surgeons.

**Case presentation:**

During a routine dissection course, an unusual origin of the double left testicular artery was observed in the cadaver of a 68-year-old Caucasian man who donated his body to the Institute of Anatomy.

**Conclusions:**

A deeper understanding of the variations of the testicular arteries is necessary for all physicians whose practice is related to the testicles and their vascular stalk.

## Introduction

The testicular arteries are known to originate from the ventrolateral aspect of the abdominal aorta and descend obliquely to the pelvic cavity. Variations in the number and course of the testicular arteries, often coexisting with variations of the other branches arising from the abdominal aorta, are reported to be less frequent than the variations of the homologous veins [1,2]. The developmentally-based variation of origin dealt mostly with the debranching from the renal artery, either main or accessory [3-7]. Although other anatomical peculiarities were related to the same cadaver, such as an early division of the right axillary artery, the presence of the axillary arch on the right side, and the evidence of the accessory renal artery of the same side of the double testicular artery, our aim was to describe, according to the literature, the rarest of the evidenced anatomical variations.

## Case presentation

**Figure 1:**
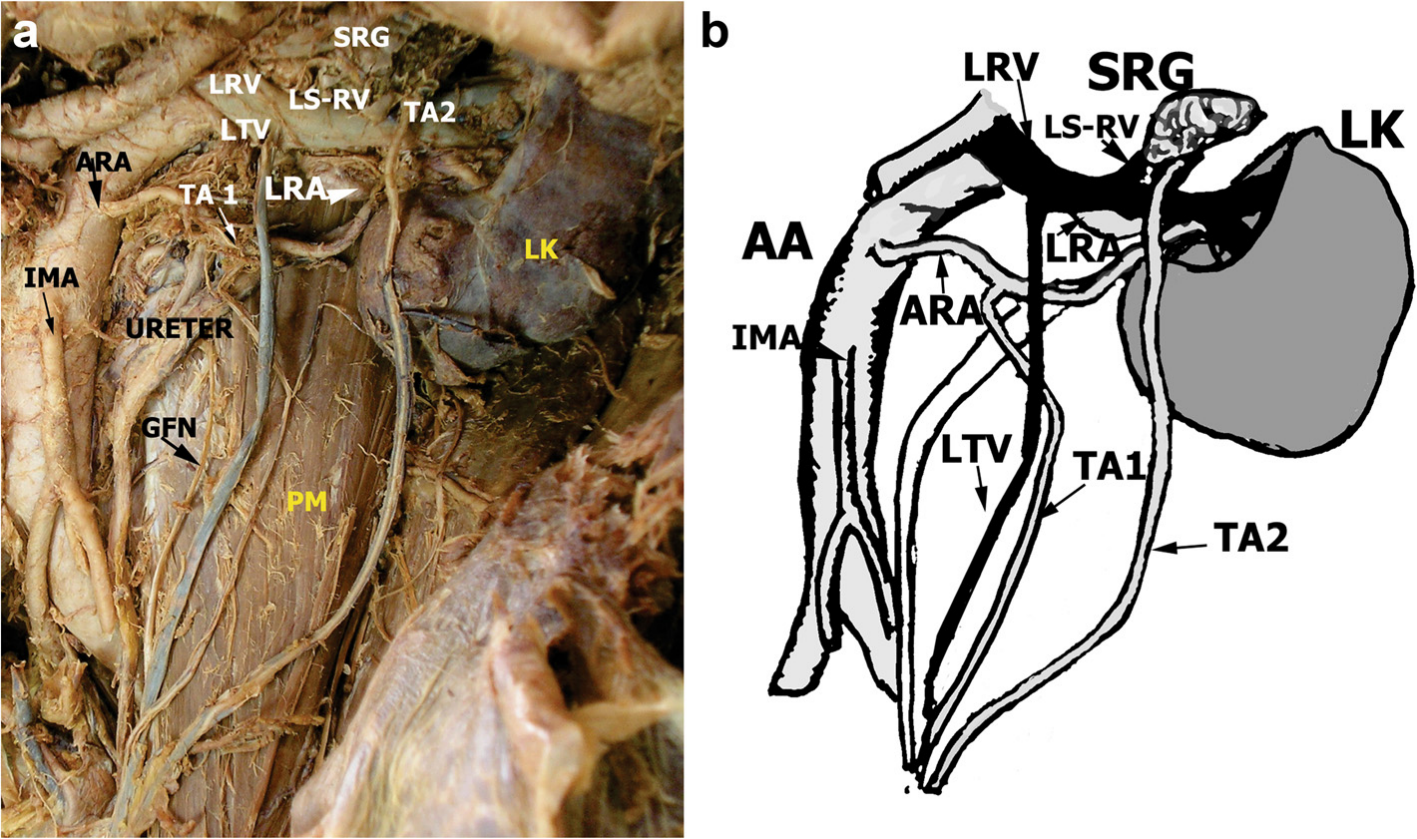
**The photo and schematic approach of the case.** TA1 and TA2. Medial and lateral testicular arteries. ARA – accessory renal artery. LRA – left renal artery. SRG – part of the suprarenal gland pierced by the lateral testicular artery (TA2). LK – left kidney. AA – abdominal aorta. IMA – inferior mesenteric artery. LS-RV – left suprarenal vein. GFN – genitofemoral nerve. PM – psoas major muscle.

During routine dissection classes, two left testicular arteries were revealed in the cadaver of a 68-year-old Caucasian man who donated his body through the body donation program: one was medial, originating from an accessory renal artery; the other was lateral, debranching from the common trunk together with the left inferior suprarenal artery. The medial artery was smaller in caliber (0.9mm) and followed the course of the regular testicular vein. The lateral left testicular artery was slightly larger (1.2mm) and pierced the tissue of the suprarenal gland immediately after debranching. The lateral artery ran in front of the left renal vein, arched laterally afterwards, ventrally crossed the inferior pole of the left kidney, and followed the lateral border of the psoas major muscle. After a short pathway on the anterior surface of the psoas major, taking the medial course, it joined the medial artery at the entrance of the funiculus spermaticus. The lateral artery was accompanied by two veins, tributaries of the left suprarenal vein. The right testicular artery had the origin and pathway described in most anatomy textbooks (Figure 1). In the man’s medical chart, no sign of any kind of urological disorder was noted, and no fertility problem was noted in his personal medical record. It was not the only anatomical variation found: a division of the axillary artery to the superficial and deep brachial artery was present in the right axilla, and on the left side an axillary arch was revealed.

## Discussion

We present a case of double left testicular artery in which one branch originated from the left renal accessory artery and the other branch arose from the common trunk with the left inferior suprarenal artery. The special feature of this case is that the testicular artery on the left side is double, and none of the two arterial vessels arose from the abdominal aorta. Yet cases concerning the origin of the double left testicular artery from the common trunk with the superior adrenal and the inferior mesenteric artery have been presented [8]. The prevalence of variations upon gonadal arteries was reported to be 16 out of 180 specimens obtained from human fetuses and to be more frequent in men than in women [9]. The variations of the testicular artery could be divided in two groups: (a) variations in the branching level, regarding the vertebral column, or arising locus of the renal arteries (above or below the branching level of the renal arteries) [6] and (b) variation in number, origin, and course; this variation appeared to be more frequent on the right side [9,10]. Also, common origin with the inferior suprarenal artery has been reported [1,8]. Testicular arteries were documented to rise from lumbar, renal, accessory renal, middle, and, occasionally, superior suprarenal arteries [4,7,11]. In a recent case presentation, Paraskevas *et al*. [12] (2011) described a high origin (above the expected level of branching) of the left testicular artery in a common trunk with the inferior phrenic artery. The bilateral branching of the ovarian arteries from the accessory renal arteries has also been revealed [13]. Most of the variations, including those described in this paper, have their roots in the embryology of the testes and the contemporaneous blood supply of each phase of development: mesonephros itself was described to have irrigation from nine mesonephric arteries: superior or cranial, middle, and inferior or caudal (three in each group). The caudal group gives rise to the testicular arteries, whereas the middle forms renal arterial vessels [3]. In our case, both vessels on the left side seemed to originate from the middle group. Double testicular arteries have been obtained in two out of 32 overlooked specimens of human fetuses [14], and both of the testes had an abdominal localization. Whether the arteries supplying undescended testes have variations in number and origin remains unclear, although a different anastomotic network is present in the undescended testicles [15]. A deeper understanding of these variations and their special relationships to adjacent vessels is especially significant in avoiding sometimes serious complications in clinical operation and other procedures, such as the Fowler-Stephens technique [16], and in recognizing the causes of genital disorders, such as cryptorchidism, and their adequate treatment in order to preserve the functionality of the undescended testis.

## Conclusions

Although many kinds of variations of the origin, direction, and pattern of the testicular arteries have been described, this is a rare case of double testicular artery on the left side. Neither of the two branches originated from the abdominal aorta but from the left inferior suprarenal artery and from the left renal artery. The awareness of such variation is important for all surgeons whose interest is related to the testicular blood vessels or, generally, blood vessels of the retroperitoneum.

## Consent

Written informed consent was obtained from the patient’s next of kin for publication of this manuscript and accompanying images. A copy of the written consent is available for review by the Editor-in-Chief of this journal.

## Competing interests

The authors declare that they have no competing interests.

## Authors’ contributions

In the order listed on the title page, BF prepared the manuscript for publication. LS, along with his students, performed anatomical dissection. BF edited the manuscript and prepared the final version for submission. All authors read and approved the final manuscript.
